# The Complex Subtype-Dependent Role of Connexin 43 (*GJA1*) in Breast Cancer

**DOI:** 10.3390/ijms19030693

**Published:** 2018-02-28

**Authors:** Mélanie Busby, Michael T. Hallett, Isabelle Plante

**Affiliations:** 1 INRS-Institut Armand-Frappier, Laval, QC H7V 1B7, Canada; melanie.busby@inrs.iaf.ca; 2 Centre for Structural and Functional Genomics, Department of Biology, Concordia University, Montreal, QC H4B 1R6, Canada; michael.hallett@concordia.ca

**Keywords:** gap junctions, Connexin 43 (Cx43), breast cancer, survival analysis, intrinsic subtype

## Abstract

Gap junction transmembrane channels allow the transfer of small molecules between the cytoplasm of adjacent cells. They are formed by proteins named connexins (Cxs) that have long been considered as a tumor suppressor. This widespread view has been challenged by recent studies suggesting that the role of Connexin 43 (Cx43) in cancer is tissue- and stage-specific and can even promote tumor progression. High throughput profiling of invasive breast cancer has allowed for the construction of subtyping schemes that partition patients into at least four distinct intrinsic subtypes. This study characterizes Cx43 expression during cancer progression with each of the tumor subtypes using a compendium of publicly available gene expression data. In particular, we show that Cx43 expression depends greatly on intrinsic subtype. Tumor grade also co-varies with patient subtype, resulting in Cx43 co-expression with grade in a subtype-dependent manner. Better survival was associated with a high expression of Cx43 in unstratified and luminal tumors but with a low expression in Her2e subtype. A better understanding of Cx43 regulation in a subtype-dependent manner is needed to clarify the context in which Cx43 is associated with tumor suppression or cancer progression.

## 1. Introduction

Connexin 43 (Cx43), a protein encoded by the Gap Junction protein alpha 1 gene (*GJA1*), forms gap junction transmembrane channels facilitating communication between the cytoplasm of two adjacent cells. Small molecules, including metabolites, second messengers and electrical signals pass through these channels in a process called Gap Junction Intercellular Communication (GJIC). Cx43 transcription is thought to be regulated both by transcription factors and by epigenetic mechanisms [1], but is also regulated at the protein level by post-transcriptional modifications, trafficking to and from the plasma membrane and gating of the channels [2].

The breast epithelium is composed of two layers of cells: an inner layer of luminal cells surrounded by an outer layer of basal cells, composed mainly of myoepithelial cells but also comprising stem and progenitor cell populations [3]. It is well established that Cx43 is expressed mainly in the basal layer; however, a few studies showed Cx43 expression in luminal cells [4,5,6]. A study using transmission electron microscopy reported gap junctions to be present between the basal and the luminal layers in normal breast tissues, although the exact connexin involved was not determined [7]. A few studies have also demonstrated the expression of Cx43 in fibroblasts surrounding the breast epithelium and in endothelial cells [8,9,10].

The role of Cx43 in breast cancer is controversial. On the one hand, Cx43 has long been considered a tumor suppressor [11] with studies demonstrating it was under-expressed at the mRNA and the protein level in cancer cell lines [12,13] or aberrant localization and phosphorylation in tumors [12,13,14,15,16]. Cx43 has also been linked to the control of processes associated with breast cancer progression and metastasis such as proliferation, invasion, migration and apoptosis [17]. Moreover, it was shown in vivo and in vitro that metastatic capacity was increased in tumors cells showing a weak GJIC capacity and a lower number of gap junction plaques [18,19]. Re-expression of Cx43 in tumor cells led to reduced growth of tumors in nude mice and fewer metastases to the lungs [20,21]. Mice expressing a mutant form of Cx43 (G60S) also showed increased breast tumor metastasis to the lung [3].

On the other hand, much evidence suggested that Cx43 is involved in later stages of breast cancer progression. For instance, it has been suggested that Cx43 mediates the interaction between tumor and endothelial cells to facilitate adhesion and extravasation at secondary sites [22,23,24]. Cx43 has also been found to be expressed at higher levels in lymph node metastasis than in the corresponding primary tumor [25]. The context of expression that allows Cx43 to act as a tumor suppressor or promoter has not been elucidated and therefore precludes its targeting in breast cancer therapies [11].

Breast cancer is highly heterogeneous, with both intra- and inter-tumoral molecular variability. During the last decade, high throughput techniques have generated a body of new data in many diseases including breast cancer. Genome-wide gene expression profiling has produced classification schemes including the intrinsic subtypes consisting of luminal A (LumA), luminal B (LumB), basal-like and HER2-enriched (Her2e) tumors. Luminal tumors are generally characterized by the expression of the estrogen receptor alpha (ERα) and the progesterone receptors (PR). Most Her2e tumors harbor a genomic amplification of chromosome 17q12 that contains the erb-b2 receptor tyrosine kinase 2 gene (ERBB2/HER2). Approximately half of Her2e tumors express ERα. Basal-like tumors are often negative for ERα or PR receptors as well as for HER2 and also express basal cytokeratins [26,27].

This study aims to investigate Cx43’s ambiguous role during cancer progression with each of the breast tumor intrinsic subtypes using a compendium of publicly available gene expression data with large samples. Here, we report that Cx43 expression depends greatly on intrinsic subtype. Tumor grade also co-varies with patient subtype, resulting in Cx43 co-expression with grade in a subtype-dependent manner. Better survival was associated with a high expression of Cx43 in unstratified and luminal tumors but with a low expression in the Her2e subtype.

## 2. Results

### 2.1. *GJA1* Expression and Localization in the Breast

**Figure 1:**
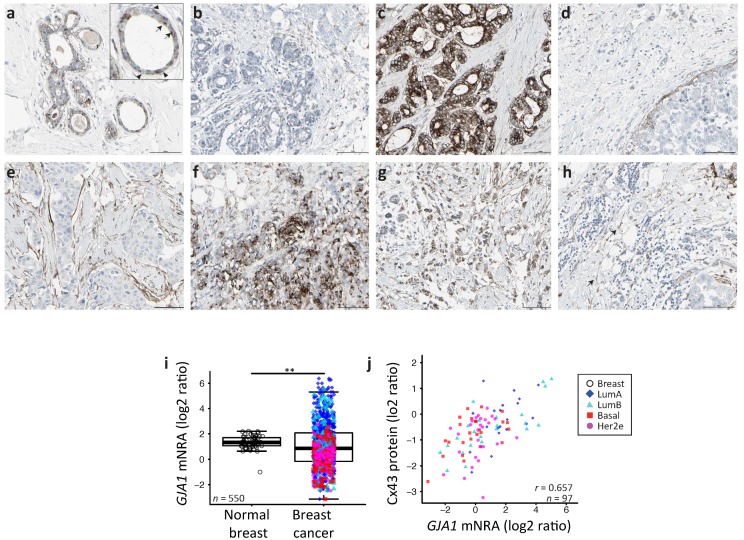
(**a**–**h**) The Human Protein Atlas normal and breast cancer tissue staining by immunohistochemistry for Connexin 43 (Cx43) (CAB010753 antibody). (**a**) Normal breast. Insert: Arrow head: myoepithelial cell’s staining; arrow: luminal cell’s staining. (**b–h**) Breast cancer tissue, (**h**) arrow: staining of spindle shaped stromal cells. Scale bar = 100 μm. (**i**) *GJA1* mRNA expression in breast tumor vs. adjacent normal breast tissue in the The Cancer Genome Atlas (TCGA) dataset. *p* value: * <0.05; ** <0.01; *** <0.001. (**j**) Scatter plot showing Cx43 protein and *GJA1* mRNA level in tumors. In the legend, “Breast” indicates adjacent normal breast tissue. Pearson’s correlation coefficient is given (r).

We first investigated the tissue localization and level of expression of Cx43 protein in human samples of both morphologically normal breast tissue and tumors using the Human Protein Atlas. This is a public database containing a large collection of normal and cancer tissue slides which have been probed with various antibodies followed by a hematoxylin counterstain [28]. Cx43 is a membrane channel and is usually considered to be expressed in the myoepithelial cell. A typical punctate staining of junctional plaques formed by Cx43 channels was observed for normal tissues. The staining could be observed in the myoepithelial layer, as expected, but also in some luminal cells (Figure 1a). Although an under-expression of Cx43 protein is observed in some of the 21 cancer samples available (Figure 1a), others show a clear over-expression, mostly in well differentiated luminal-like neoplastic cells, which did not appeared to be associated with a basal layer (Figure 1c). In other samples, Cx43 could also be seen in a layer of cells separating neoplastic tissue from stroma, although this layer sometimes adhered poorly to both adjacent compartments (Figure 1d,e). Cx43 was also observed in samples with poorly differentiated cell and tissue morphology (Figure 1f,g). Interestingly, Cx43 protein could also be found in spindle-shaped cells in the stroma (Figure 1h). Overall, some normal punctate patterns could be observed in some tumors (Figure 1d) while the majority of the samples showed either a downregulation or an aberrant cytoplasmic localization of Cx43 in tumor cells.

We next compared transcript levels of *GJA1* in breast tumor samples and in the non-cancerous adjacent tissues using microarray-based data of The Cancer Genome Atlas project (TCGA) breast invasive carcinoma cohort (BRCA) of clinical samples. We observed a far greater variance in mRNA expression in tumor samples compared to tumor adjacent morphologically normal breast tissue (Figure 1i, the Fligner–Killeen test of homogeneity of variances, *p* value < 10^−12^). We also used whole sample Cx43 protein levels obtained for 105 TCGA samples by mass spectrometry. *GJA1* mRNA and protein level are significantly correlated (Figure 1j, (Pearson correlation rho = 0.6515, *p* value < e^−13^). Our results confirm that, in breast cancer, *GJA1* is concurrently dysregulated at both the protein and the mRNA level.

### 2.2. *GJA1* Expression Varies with Breast Cancer Subtype

**Figure 2:**
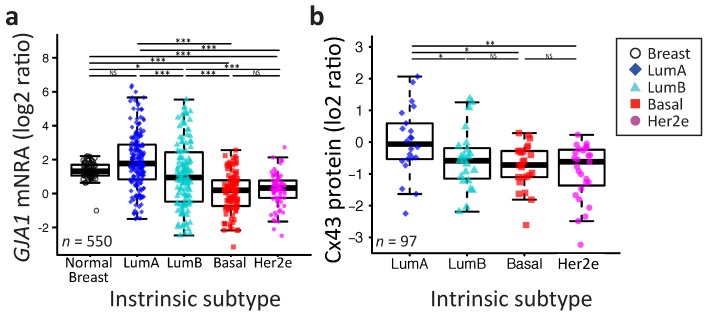
The *GJA1* expression level is more variable in breast tumor than in normal tissue and varies with subtype. (**a**) The *GJA1* mRNA level in normal breast tissue and in each tumor intrinsic subtype. (**b**) The Cx43 protein level in each intrinsic subtype. In the legend, “Breast” indicates adjacent normal breast tissue. All data are from the TCGA dataset. *p* value: * <0.05; ** <0.01; *** <0.001; NS Not statistically significant.

We then speculated that *GJA1* variability could be linked to the molecular heterogeneity of breast cancer. When we compared *GJA1* mRNA expression after stratifying patient samples by their intrinsic subtype (Pam50 by genefu [29]) (Figure 2a), the increase in variance in gene expression of tumor samples relative to normal tissue was observed in every subtype. The LumA had a mean expression level statistically indistinguishable from morphologically normal samples, but a small significant progressive decrease in expression is observed from LumB to basal and Her2e subtypes (Figure 2a).

**Figure A1:**
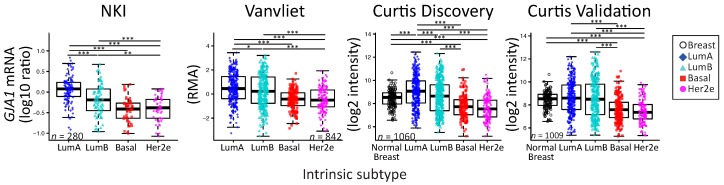
*GJA1* mRNA level in breast cancer clinical samples is dependent on breast cancer subtype in four datasets. *GJA1* mRNA level in tumors stratified by subtype in Vanvliet, NKI, Curtis Discovery and Curtis Validation datasets. In the legend, “Breast” indicates adjacent normal breast tissue. *p* value: * <0.05; ** <0.01; *** <0.001.

A similar pattern was observed in the four other datasets, although normal breast tissues were only used in TCGA and Curtis datasets (Figure A1). A similar pattern was also observed at the protein level in the TCGA dataset (Figure 2b). Together, these results suggest that the expression of *GJA1* is strongly associated with tumor subtype and is more variable in each subtype in comparison to morphologically normal tissue.

### 2.3. Somatic DNA-Level Events of *GJA1* Do Not Drive Expression Changes of *GJA1* in Breast Cancer

**Figure 3:**
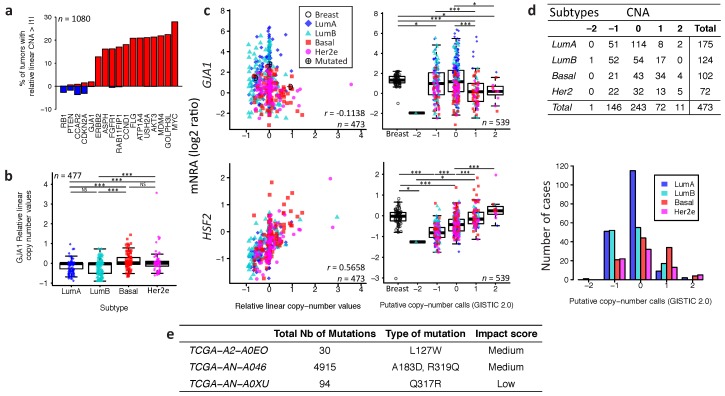
Somatic DNA-level events of *GJA1* do not drive expression changes of *GJA1* in breast cancer. (**a**) Percentage of tumors with a relative linear copy number >1 (amplification, in red) and <−1 (hemi- or homozygous deletion, in blue) for *GJA1* compared to other genes known to be altered in breast cancer. (**b**) Relative linear copy number value for each breast cancer subtype. (**c**) Somatic copy number alteration and putative copy number calls against mRNA expression for *GJA1* and *HSF2*. Copy number calls were computed by TCGA using GISTIC 2.0 (−2, Homozygous deletion; −1, Hemizygous deletion; 0, Neutral/no change; 1, Gain; 2, High level amplification). Pearson’s correlation coefficient between relative linear copy number value and mRNA expression is given *GJA1* and *HSF2*. In the legend, “Breast” indicates adjacent normal breast tissue. (**d**) Contingency table and barplot showing the distribution of copy number alteration (CNA) by subtype. Due to the small number of samples in −2 and 2 CNA, Fisher’s exact test was applied on −1, 0 and 1 CNA. (**e**) Total number and *GJA1* mutations observed in the 3 cases out of 988 patients. All data from the TCGA dataset. *p* value: * <0.05; ** <0.01; *** <0.001; NS Not statistically significant.

We next asked if underlying DNA-level somatic copy number changes in the genomic loci harboring *GJA1* influence gene expression levels. For the TCGA dataset, a few tumors had amplification or deletion of *GJA1*, compared to genes known to be amplified in breast cancer (Figure 3a).

Moreover, tumors with *GJA1* amplification did not show an increase in expression while only deep deletions reduced expression (−2 in called copy number, as shown in Figure 3c). Most luminal tumors with the highest expression of *GJA1* were found to have either a normal copy number or single deletion (Figure 3b–d). Moreover, in tumors with a *GJA1* gain or amplification, a slight but significant decrease in expression, rather than an increase, could be observed compared to normal tissues (Figure 3c). *GJA1* mRNA also weakly negatively correlated with DNA copy number (Figure 3c), suggesting that Cx43 over-expression in breast cancer is not driven by DNA amplification. To validate our procedure, we used the *HSF2* gene, a close neighbor of *GJA1* on chr6q22 which shares similar copy number in 99% of TCGA’s breast cancer cases. In contrast to what was observed for *GJA1*, *HSF2* mRNA was positively correlated with the copy number of its own gene (Figure 3c). Moreover, somatic point mutation data showed that, in the TCGA cohort, only three breast cancer patients out of 977 harbored at least one *GJA1* mutation, accounting for 0.31% of the tumors (Figure 3e). Only one tumor with an extremely high number of total mutations (TCGA-AN-A046) was found to have both a *GJA1* mutation and a slightly higher expression of the gene (2.76) compared to normal range (−1.01 to 2.20) (Figure 3c,e). Together, these results argue that loss or amplification of the *GJA1* gene likely does not dictate mRNA and protein dysregulation in breast cancer.

### 2.4. *GJA1* Level Is Dependent on Hormonal Receptor Status

Because Cx43 level varies through the mammary gland development and the reproductive cycle, it has been suggested that it could be regulated by hormones, similar to what has been observed in other tissues [4,30,31,32]. We thus next investigated whether the *GJA1* mRNA level was directly linked with hormonal receptors status.

**Figure 4:**
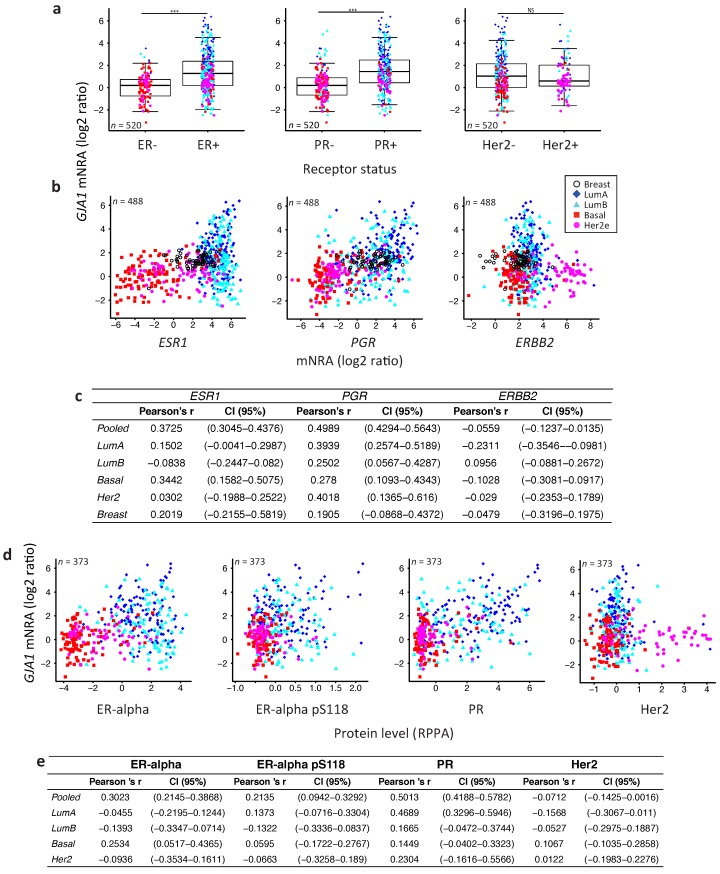
Depending on the receptor status, *GJA1* is associated with different mRNA levels in clinical samples. (**a**) Expression of *GJA1* mRNA stratified by estrogen receptor alpha (ERα), progesterone receptor (PR) and erb-b2 receptor tyrosine kinase 2 (ERBB2/Her2) status in the TCGA dataset. *p* value: * <0.05; ** <0.01; *** <0.001; NS Not statistically significant. (**b**) Plot of *GJA1* vs. *ESR1*, *PGR* and *ERBB2* mRNA (microarray) level in each subtype and in normal breast tissue. In the legend, “Breast” indicates adjacent normal breast tissue. (**c**) Bootstrapped correlations between *ESR1*, *PGR* or *ERBB2* and *GJA1* mRNA level either in pooled breast cancer tumors or in individual breast cancer intrinsic subtypes and in normal breast tissue. (**d**) Plot of *GJA1* mRNA vs. ER-alpha, ER-alpha pS118, PR or HER2 protein level assessed by reverse phase protein assay (RPPA). (**e**) Bootstrapped correlations between *GJA1* mRNA and ER-alpha, ER-alpha pS118, PR or HER2 protein level (RPPA). All data are from TCGA’s BRCA dataset.

**Figure A2:**
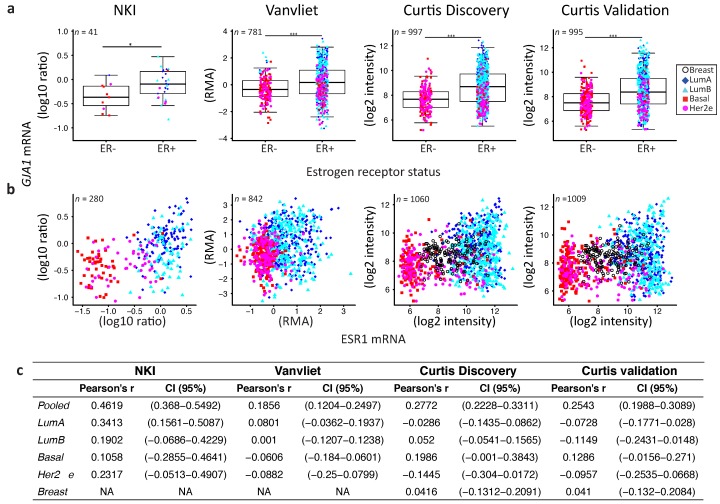
*GJA1* mRNA level in breast cancer clinical samples is higher in ER-positive tumors in four datasets. (**a**) *GJA1* mRNA level in breast tumors stratified by estrogen receptor status determined as described in Material and Methods. *p* value: * <0.05; ** <0.01; *** <0.001. (**b**) Expression of *GJA1* vs. estrogen receptor alpha (*ESR1*) mRNA in each subtype and in normal breast tissue. (**c**) Bootstrapped correlations between *ESR1* and *GJA1* mRNA level either in pooled breast cancer tumors or in individual intrinsic subtypes and in normal breast tissue. Data from Vanvliet, NKI, Curtis Discovery and Curtis Validation datasets.

**Figure A3:**
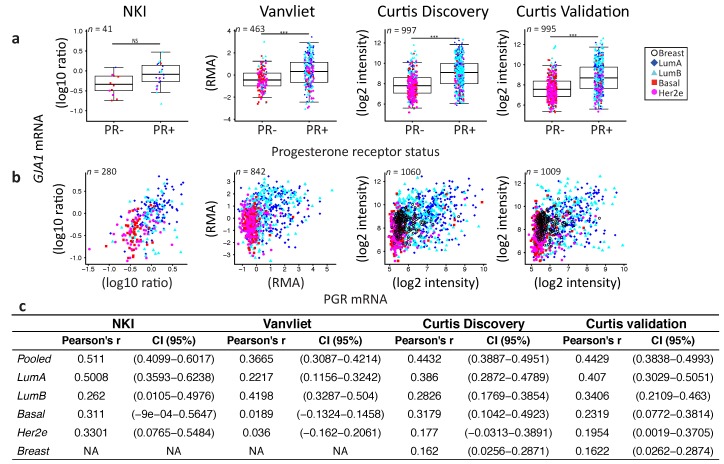
*GJA1* mRNA level in breast cancer clinical samples increases with *PGR* expression in four datasets. (**a**) *GJA1* mRNA level in breast tumors stratified by PR status determined as described in Material and Methods. In the legend, ”“Breast” indicates adjacent normal breast tissue. *p* value: * <0.05; ** <0.01; *** <0.001; NS Not statistically significant. (**b**) Expression of *GJA1* vs. progesterone receptor (PGR) mRNA in each subtype and in normal breast tissue. (**c**) Bootstrapped correlations between *PGR* and *GJA1* mRNA level either in pooled breast tumors or in individual breast cancer intrinsic subtypes. Data from Vanvliet, NKI, Curtis Discovery and Curtis Validation datasets.

Consistent with the subtype-specific expression of Cx43, ERα- or PR-positive breast tumors had a significantly higher expression of *GJA1* mRNA compared to ERα- or PR-negative tumors (Figure 4a, Figure A1, Figure A2 and Figure A3). Results were similar for all five datasets, except for PR in the NKI dataset where the low number of samples did not allow statistical significance to be reached (Figure 4a, Figure A2a and Figure A3a). However, there were no strong correlations between *GJA1* expression and *ESR1* mRNA, with total protein level (ERα), or with the activated form of ERα phosphorylated on serine 118 (ERα_pS118) (Figure 4b–e and Figure A1b,c). While only weak correlations were observed in most individual subtypes, a stronger correlation between *GJA1* and *ESR1* mRNA and protein was observed when the tumors were pooled (Figure 4b–d). As expected, *ESR1* mRNA was better correlated with total ERα (Pearson’s rho = 0.9011, Spearman’s rho = 0.8969) than with ERα_pS118 proteins (Pearson’s rho = 0.5459, Spearman’s rho = 0.6407).

Stronger correlations between *PGR* mRNA and protein levels and *GJA1* mRNA levels were observed, not only in unstratified (pooled) analysis, but also in individual subtypes within most datasets (Figure 4b–e and Figure A1b,c). This association was stronger in cancer samples than in normal breast tissues in all datasets for which normal tissues were available. Similar to ERα, total PR protein was well correlated with *PGR* mRNA (Pearson’ rho = 0.8593 and Spearman’s rho = 0.8723).

**Figure A4:**
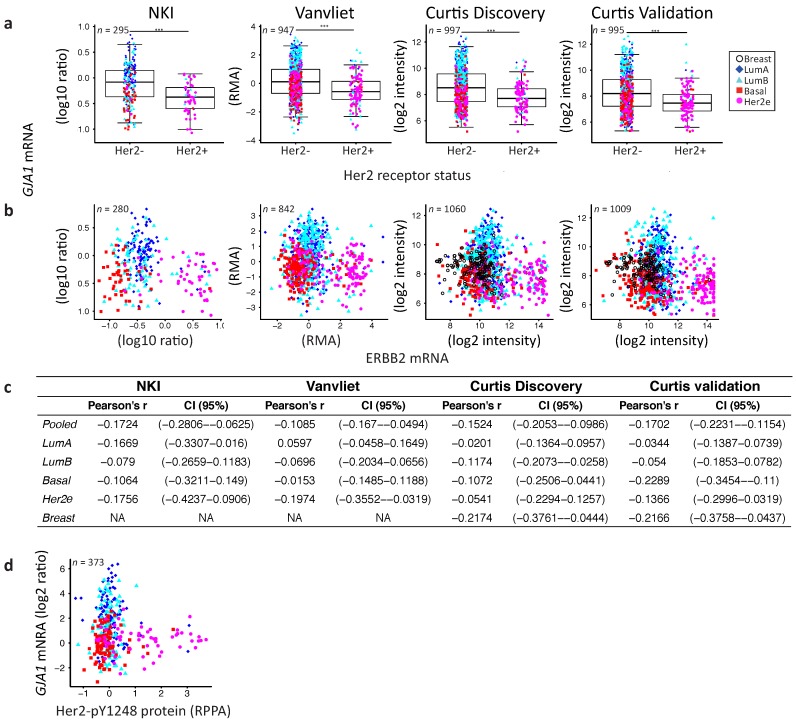
*GJA1* mRNA level in breast cancer clinical samples is lower in HER2-positive breast tumors in four datasets. (**a**) *GJA1* mRNA level in breast tumors stratified by HER2 status determined as described in Material and Methods. In the legend, “Breast” indicates adjacent normal breast tissue. *p* value: * <0.05; ** <0.01; *** <0.001. (**b**) Expression of *GJA1* vs. *ESR1* mRNA in each subtype and in normal breast tissue. (**c**) Bootstrapped correlations between *ERBB2* and *GJA1* mRNA level either in pooled breast cancer tumors or in individual intrinsic subtypes and in normal breast tissue. Data from Vanvliet, NKI, Curtis Discovery and Curtis validation datasets. (**d**) *GJA1* mRNA vs. pY1248 phosphorylated form of HER2 protein level determined by RPPA in breast tumors from the TCGA dataset.

Tumors positive for the HER2 receptor by histochemistry (TCGA dataset) did not express significantly different levels of Cx43 mRNA. However, when HER2 status was given by HER2 amplicon probes or HER2 mRNA expression (Vanvliet, NKI and Curtis datasets), HER2+ tumors had a significantly lower level of *GJA1*. No direct correlation was observed between *GJA1* and the HER2 (ERBB2) mRNA (Figure 4a–e and Figure A4a–d). A good correlation was observed between HER2 mRNA and total HER2 protein level (Pearson’s rho = 0.8344, Spearman’s rho 0.68634). The correlation between HER2 protein (HER2 and HER2_pY1248 activated form) and *GJA1* mRNA was not stronger than that observed for HER2 mRNA (Figure 4b–e and Figure A3d).

Together, the significant differences observed in *GJA1* mRNA level in individual subtypes and with receptor status suggest that *GJA1* level is dependent on the molecular context provided by such subtypes. In addition, *GJA1* does not vary directly with *ESR1* and HER2 mRNA and protein levels but shows a stronger correlation with *PGR* mRNA and PR protein in tumor samples.

### 2.5. *GJA1* mRNA Is Dysregulated at the Early Stages of Breast Cancer and Is Reduced with Grade When Tumors Are Pooled

**Figure 5:**
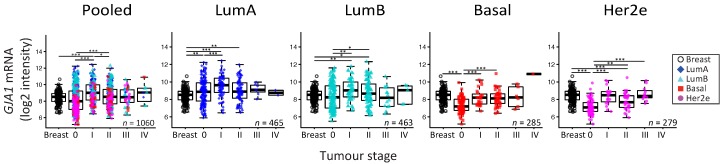
*GJA1* mRNA level is dysregulated in clinical samples at the early stages of breast cancer. *GJA1* mRNA level for each tumor stage and for normal breast in the Curtis Discovery dataset, either in pooled breast tumors or stratified by intrinsic subtype. In the legend, “Breast” indicates adjacent normal breast tissue. *p* value: * <0.05; ** <0.01; *** <0.001.

**Figure A5:**
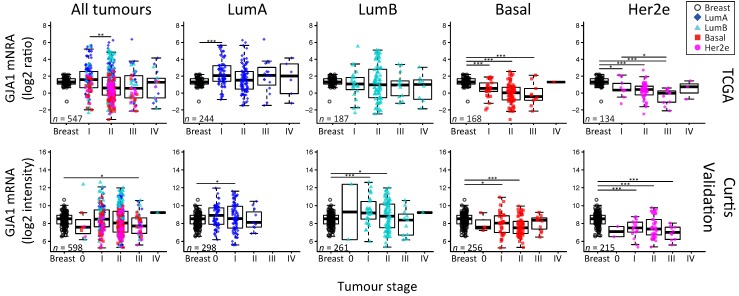
*GJA1* mRNA level is dysregulated in the early stages of breast cancer in clinical samples. *GJA1* mRNA level for each tumor stage either in pooled breast tumors or stratified by intrinsic subtype. Data from TCGA and Curtis Validation datasets. In the legend, “Breast” indicates adjacent normal breast tissue. *p* value: * <0.05; ** <0.01; *** <0.001.

To reconcile evidence supporting both tumor-suppressive and -promoting roles, it has been suggested that Cx43 function could depend on tissue type or evolve with tumor stage [11]. We therefore investigated whether *GJA1* expression in primary breast tumor changed with stage and grade at the mRNA level in breast cancer. Since grade/stage are strongly associated with subtype, we first stratified our cohorts by intrinsic subtype. We used the Curtis dataset, as *GJA1* expression was available for invasive tumors (stages 0 to IV) and “normal” adjacent tissue for numerous samples. A significant dysregulation of *GJA1* expression occurred at the early stages in all breast cancer subtypes, although both over-expression and downregulation could be observed (Figure 5 and Figure A5). Most of the *GJA1* over-expressing luminal tumors were found to be of low stage (0–II). However, a reduction was observed in early stage basal-like and Her2e tumors (Figure 5 and Figure A5). A significant increase in *GJA1* was also observed in the invasive stage I compared with stage 0 in all subtypes (Figure 5).

**Figure 6:**
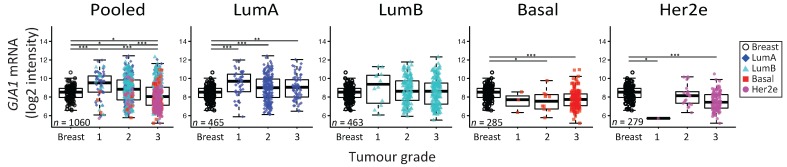
*GJA1* mRNA level is downregulated with grade in clinical samples only in pooled tumors but not in individual subtypes. *GJA1* mRNA level for each tumor grade in the Curtis Discovery dataset, either in pooled breast tumors or stratified by intrinsic subtype. In the legend, “Breast” indicates adjacent normal breast tissue. *p* value: * <0.05; ** <0.01; *** <0.001.

**Figure A6:**
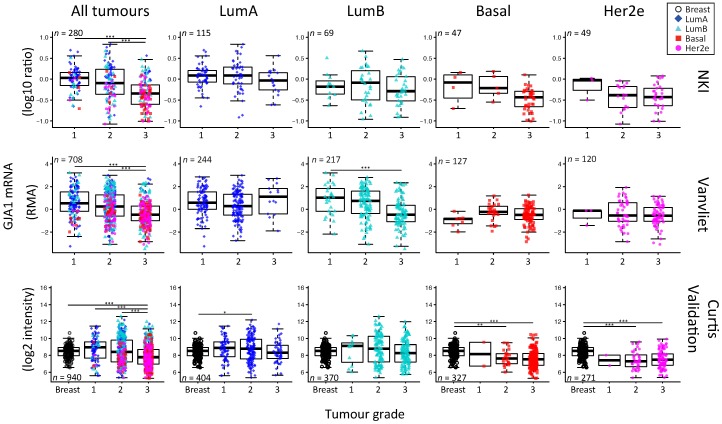
*GJA1* mRNA level is reduced with grade in breast cancer clinical samples but only in pooled tumors. *GJA1* mRNA level for each tumor grade, in Vanvliet, NKI and Curtis Validation datasets, either in pooled breast tumors or stratified by intrinsic subtype. In the legend, “Breast” indicates adjacent normal breast tissue. *p* value: * <0.05; ** <0.01; *** <0.001.

We then investigated whether or not the expression of *GJA1* could be linked to tumors’ grade. Our analysis revealed that *GJA1* mRNA expression was significantly decreased with grade when all tumors were pooled, but not when they were stratified by intrinsic subtype (Figure 6 and Figure A6). A significant decrease in *GJA1* with grades in LumB tumors could be observed only in the Vanvliet’s dataset but not in other datasets analyzed (Figure 6 and Figure A6).

Interestingly, basal and Her2e tumors, which express a low level of *GJA1* (Figure 2), account for an important proportion of grade 3 tumors, thus reducing the mean *GJA1* expression for this grade (Figure 6). Moreover, grade 1 tumors are mostly luminal A and B, with a subset of *GJA1* over-expressing tumors, introducing an upward bias in this grade. Grade 2 tumors consist of a more balanced mix of all the subtypes (Figure 2 and Figure 6). These results suggest that an observed reduction in *GJA1* with grade in pooled tumors is likely a bias induced by the pooling of the tumors’ subtypes.

### 2.6. In Her2e Breast Tumors, a Low Expression of *GJA1* Is Associated with a Better Prognosis

**Figure 7:**
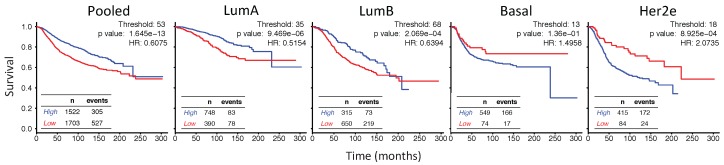
*GJA1* is associated with a diverging outcome depending on breast cancer subtype. The Kaplan–Meyer plots show survival curves for patients with breast tumors expressing either high (blue) or low (red) levels of *GJA1* mRNA in pooled tumors or in individual intrinsic subtypes. TCGA, NKI, Vanvliet and both Curtis datasets were aggregated for the analysis. The best cutoff was determined as the percentile lending the lowest log rank test *p* value (Figure A12) and was 53 in Pooled tumors, 35 in luminal A (LumA) tumors, 68 in luminal B (LumB), 13 in Basal and 18 in Her2e tumors.

**Figure A7:**
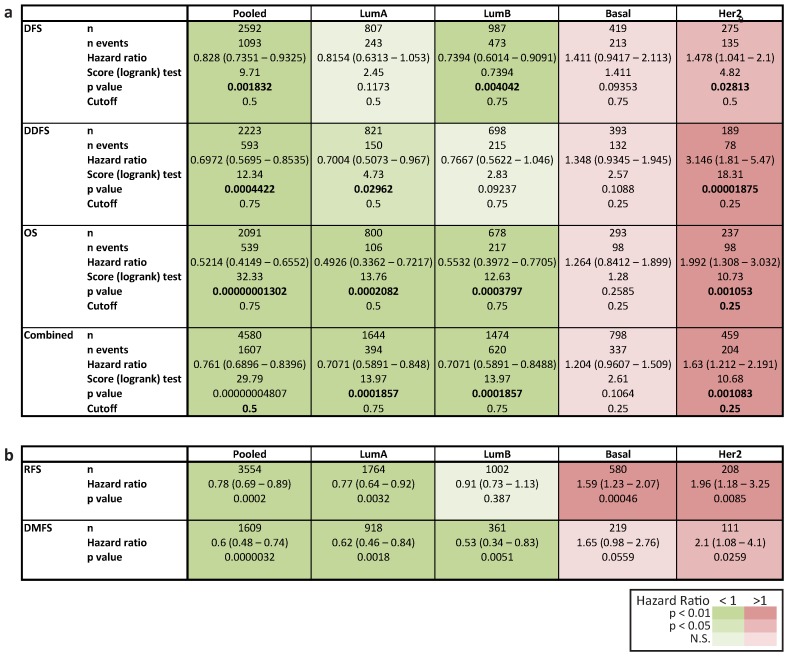
*GJA1* is associated with different outcomes depending on the breast cancer subtype. (**a**) BreastMark’s; and (**b**) KMPlotter’s web interface were used to complete the survival analysis for *GJA1* expression. Disease-Free Survival (DFS), Distant Disease-Free survival (DDFS), Overall Survival (OS) and Combined Survival (Combined) are given either for pooled tumors or for individual breast cancer subtypes in Breast Mark (**a**). Relapse-Free Survival (RFS) and Distant Metastasis-Free survival are given for *GJA1* probe 201667_at in KMPlotter (**b**). The best cutoff was determined manually for BreastMark (25, 50 or 75 percentile) based on *p* value and logrank score and automatically for KMPlotter.

To gain further insight into the role of Cx43 in breast cancer, we analyzed how the level of *GJA1* mRNA expression in each subtype was associated with outcome. Observations that Cx43 expression was associated with a worse prognostic in ER-negative [33] and Her2e [34] tumors have been previously reported using the web-based platform KMPlotter [35] while ER-positive tumors had a better prognosis [33]. Investigating further the results of BreastMark and KMPlotter Web platforms, survival analysis showed that pooled and luminal tumors with high levels of *GJA1* mRNA were associated with a better prognostic (hazard ratio < 1), although results were not always statistically significant (Figure 7 and Figure A7a,b). Conversely, basal-like and Her2e tumors followed an opposite trend (hazard ratio > 1), with high expression of *GJA1* strongly associated with a worse prognosis in the Her2e subtype (Figure 7 and Figure A7a,b).

**Figure A8:**
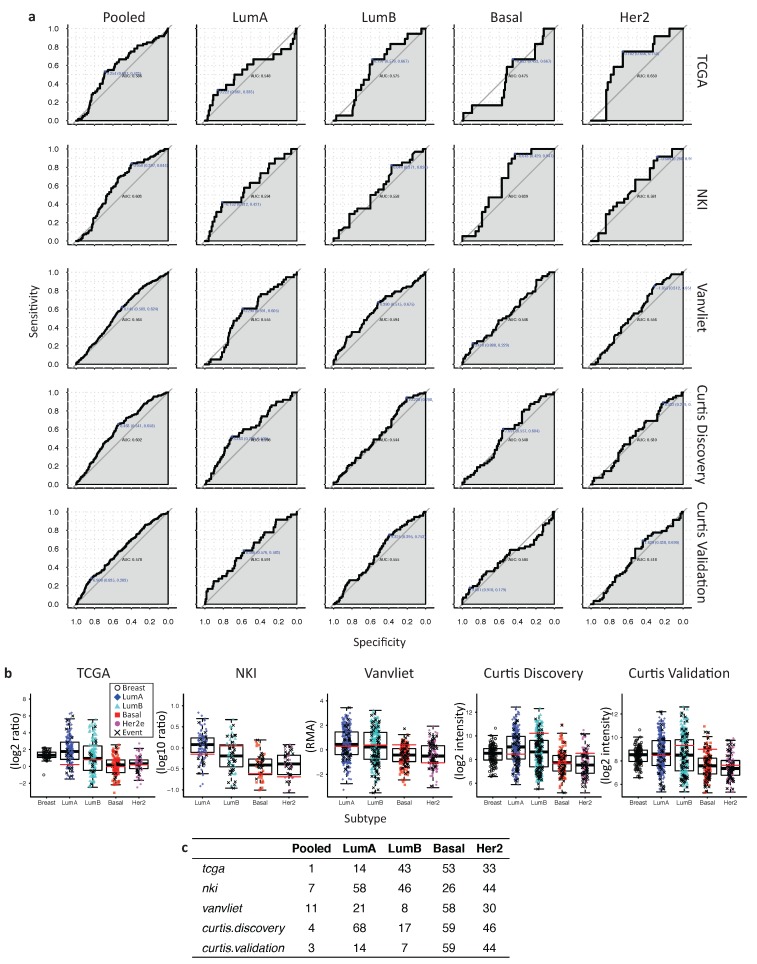
(**a**) Determination of the best cutoff for *GJA1* using ROC curves in individual subtypes of breast cancer. ROC curves evaluating the sensitivity vs. the specificity of *GJA1* as a marker of prognostic (occurrence of events in five years after tumor removal) in pooled tumors and each individual subtype of breast cancer of five datasets (TCGA, NKI, Vanvliet and Curtis discovery and Curtis validation). AUC, Area under the curve. The best cutoff is given in blue and is used in a subsequent survival analysis in Figure A9. (**b**) Expression of *GJA1* in pooled tumors and individual subtypes in five datasets. The red lines indicate the cutoff as determined by the ROC curves. In the legend, “Breast” indicates adjacent normal breast tissue. X indicates tumors with subsequent recurrence events. (**c**) Rank of the *GJA1* probe in pooled tumors and each individual subtype for five datasets. Rank given in percentile of the area under the ROC curve compared to all the other probes.

**Figure A9:**
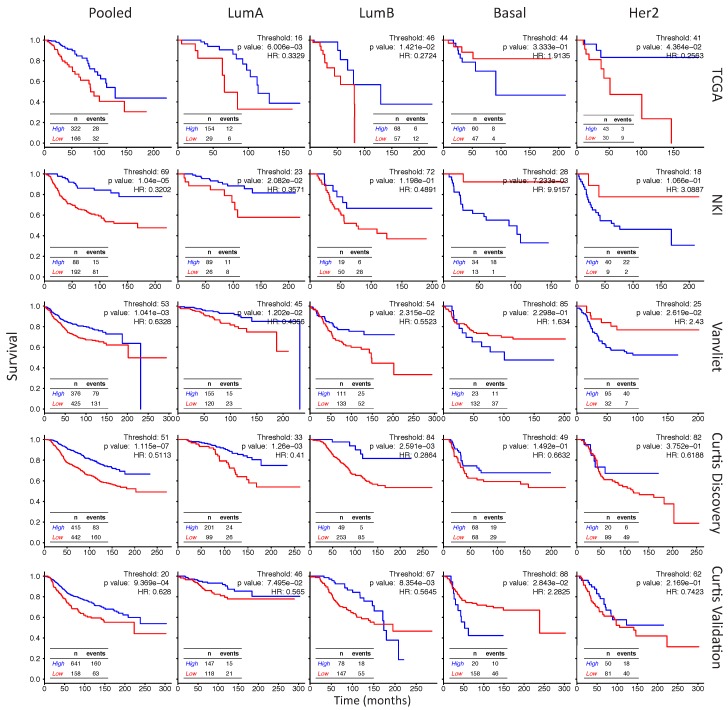
Survival analysis of *GJA1* in individual subtypes of breast cancer with cutoff selected according to the ROC curve. Kaplan–Meyer plots show survival curves for patients with breast tumors expressing either high (blue) or low (red) levels of *GJA1* mRNA in pooled tumors or in individual intrinsic subtypes. TCGA, NKI, Vanvliet and both Curtis datasets were used for the analysis. The best cutoff was determined from ROC curves as in Figure A8.

**Figure A10:**
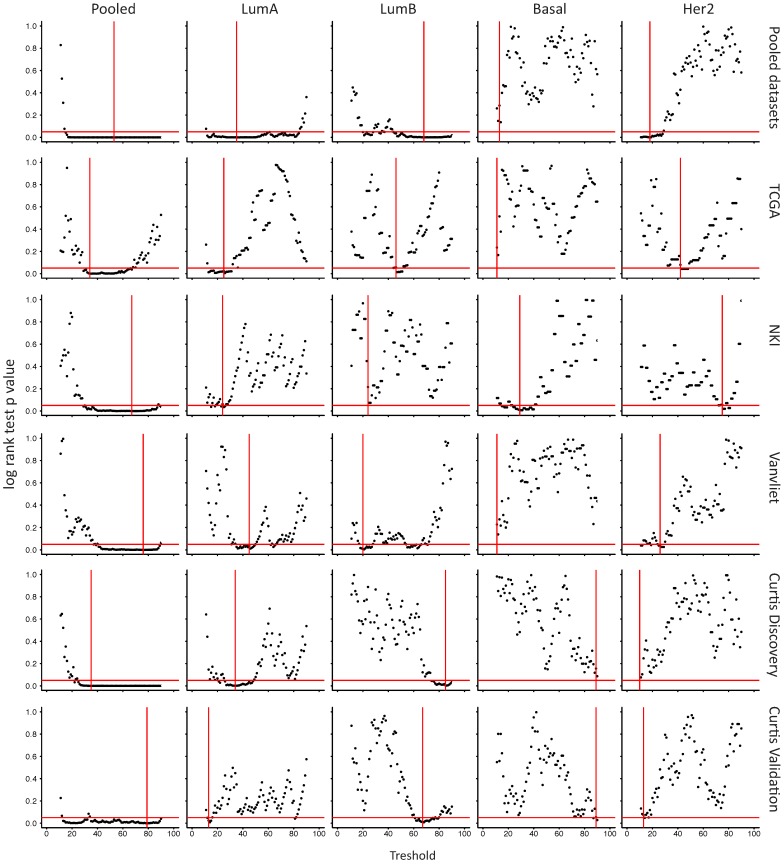
Determination of the best cutoff for *GJA1* using the log rank test *p* value in individual subtypes of breast cancer. Graph of the *p* value of the log rank test to compare the survival of patients with Cx43 expressed at high or low levels according to varying thresholds (10–90 percentile). Horizontal red lines indicate 0.05 *p* value. Vertical red lines indicate the threshold with the lowest *p* value for the log rank test, used for subsequent survival analysis in Figure 7 and Figure A12. Results given for pooled tumors or individual breast cancer subtypes in our five datasets (TCGA, NKI, Vanvliet and Curtis discovery and Curtis validation) as well as in aggregated datasets (Pooled datasets).

Since the aggregation of several datasets in BreastMark and KMPlotter platforms could lead to artifacts in survival analysis, we went further by performing our own survival analysis for each subtypes for either aggregated (Figure 7) or individual datasets (Figure A9 and Figure A12) following the determination of the best cutoff either by the receiver operating characteristic (ROC) curve (Figure A8a–c) or by the smallest *p* value of the log rank test for different thresholds (10–90) (Figure A10).

ROC curves have shown that the highest area Under the curve (AUC) for *GJA1* was obtained when tumors were pooled (Figure A8a) and *GJA1* was then ranked, at worst, in the eleven first percentiles when compared to all the probes present in the five datasets (Figure A8c). The log rank test was highly significant for all the analyses (Figure 7, Figure A9 and Figure A12) and for a vast range of cutoffs (Figure A10c), suggesting that *GJA1* has the greatest discriminating power when cohorts are unstratified. This is in line with a differential expression of *GJA1* in luminal vs. basal and Her2e tumors that also have diverging prognostics (Figure 2a).

**Figure A11:**
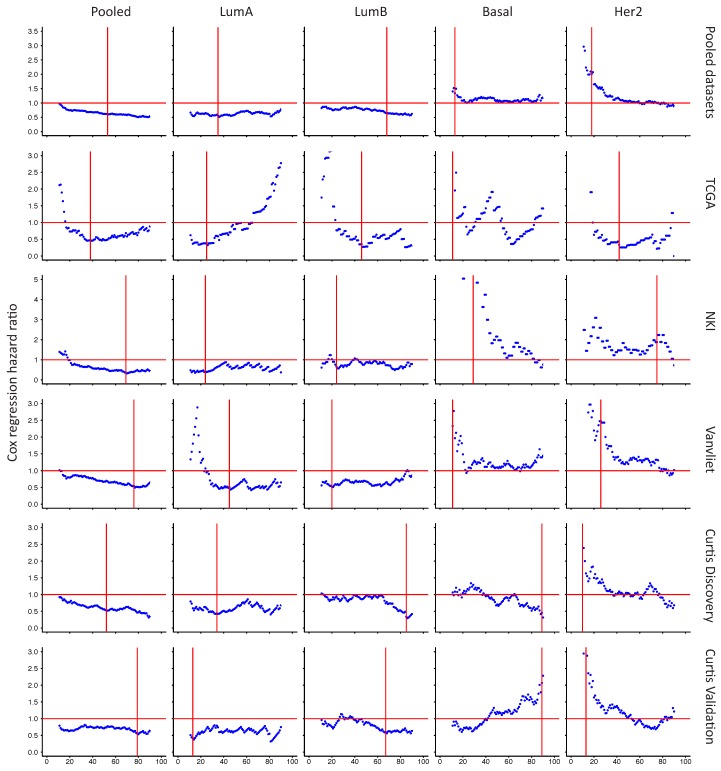
Cox regression hazard ratio associated with *GJA1* for different thresholds. Graph of the Cox regression hazard ratio to compare the survival of patients with Cx43 expressed at high or low levels according to varying thresholds (10–90 percentile). Horizontal red lines indicate a neutral hazard ratio of 1. Vertical red lines indicate the threshold with the lowest log rank test *p* value as determined in Figure A10, used for subsequent survival analysis. Results given for pooled tumors or individual breast cancer subtypes in our five datasets (TCGA, NKI, Vanvliet and Curtis discovery and Curtis validation) as well as in aggregated datasets (Pooled datasets).

**Figure A12:**
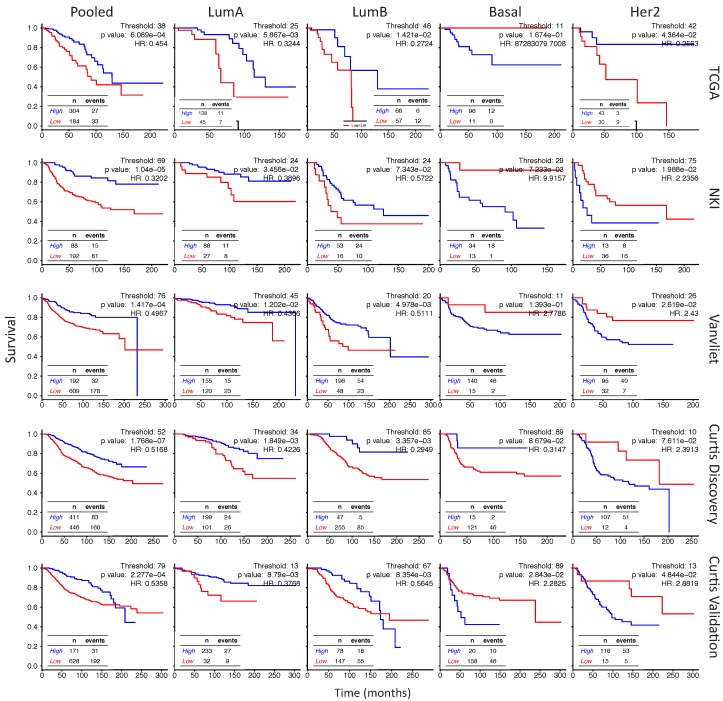
Survival analysis of *GJA1* in individual subtypes of breast cancer with the cutoff selected according to the log rank test *p* value. The Kaplan–Meyer plot shows survival curves for patients with breast tumors expressing either high (blue) or low (red) levels of *GJA1* mRNA in pooled tumors or in individual intrinsic subtypes. TCGA, NKI, Vanvliet and both Curtis datasets were used separately for the analysis. The best cutoff was determined as the percentile lending the lowest log rank test *p* value as in Figure A10.

When analyzing individual subtypes, a high expression was also significantly associated with a better prognosis in all analyses for LumA and for most analyses for LumB tumors (Figure 7, Figure A9 and Figure A12). However, survival curves in most analyses as well as the hazard ratio for a wide range of cutoffs (Figure A11) showed that this tendency is reversed in Basal and Her2e tumors where *GJA1* is mostly associated with a worse prognosis. This result was most significant in Her2e tumors, especially with smaller cutoffs while significance was rarely reached for Basal tumors.

However, *GJA1* ROC curves showed that *GJA1* did not consistently identify bad prognosis tumors with a high specificity and sensitivity (Figure A8a). These results suggest that although stratifying tumors revealed that the role of *GJA1* possibly differs in different breast cancer subtypes, *GJA1* should not be used as a clinical marker. These results also highlight once again how analyses using pooled tumor subtypes might induce biases and hide diverging results that are subtype-specific.

## 3. Discussion

Traditionally, Cx43 was considered as a tumor suppressor in the breast, with many studies reporting decreased Cx43 expression in tumor compared to normal breast tissue via both in vivo and in vitro studies [3,12,13,18,19,20,21]. However, other studies contradict these findings [8,22,23,24,25]. This recent evidence has cast doubt on Cxs tumor’s suppressive role, suggesting that the Cxs function in cancer was tissue- and tumor stage-dependent [11,17]. At least four different subtypes of breast cancer have been identified, each having unique molecular profiles, responses to treatment and prognostics. Our evidence suggests that the role of Cx43 is dependent on subtype.

### 3.1. Cx43 Expression Is Dysregulated in Breast Cancer

Early studies first showed a dramatic downregulation of *GJA1* at the mRNA and the protein level in breast cancer cell lines as well as in rat and human breast tumors [12,13]. Conversely, other studies showed an increase in a subset of tumors [15]. Most of these studies analyzed a limited number of samples and were conducted either prior to the intrinsic subtype classification of breast cancer or did not use such classification. Our results, with several large cohorts of breast cancer clinical samples, reconcile these contradictory data by demonstrating that the observed dysregulation can involve both he increased and decreased expression of the Cx43 protein and mRNA. These observations are consistent with more recent reports at the protein level [15,16,25,36].

### 3.2. Dysregulation of Cx43 Is Linked to Hormonal Receptor Status and Tumor Subtype

Our results showed that the expression of Cx43 in breast tumors was lower in basal and Her2e than in normal tissues and that Cx43 levels vary greatly within luminal subtypes. This subtype-dependent expression was also shown by more recent studies, the result of which also support a higher expression of Cx43 mRNA and protein in luminal tumors than in basal-like and Her2e subtypes [36,37]. Because the intrinsic subtypes are characterized by, among others, hormonal receptor status, we wanted to evaluate whether a functional link could be captured in whole-tumor expression profiles between Cx43 and ERα, PR or HER2. Whole-tumor expression has been used by others, both to assess the content of specific cell types in samples and to decipher functional links between genes [38,39]. Using this method, we showed that *GJA1* mRNA increases in a subset of ERα- and PR-positive tumors and in the luminal subtypes, which are largely ERα- and PR-positive. These results were not surprising as much evidence supports a link between Cx43 and hormones in breast tissue [32] and in other tissues [40,41,42,43]. *GJA1* is also expressed at lower levels when HER2 status is positive and within the Her2e breast cancer subtype, except in the TCGA dataset. In an early study, it was reported that Cx43 gap junctions were dramatically reduced in breast tumors, and that this reduction was considered to occur regardless of ERα, PR or HER2 status [12]. More recent studies have reported that Cx43 protein expression correlated positively with PR and ERα status [44,45] and negatively with HER2 protein expression [45]. However, Conklin et al. reported that no correlation was observed between Cx43 and HER2 protein in tissue microarrays [44].

Our results suggest a direct relationship between *GJA1* and PR expression in breast cancer samples. Our analysis shows that *GJA1* level correlates with PR mRNA and protein in several subtypes. These results suggest that either PR or *GJA1* levels are dependent on the relative amount of some cell types co-expressing both genes, or that a functional link exists in the regulation of these genes in the same cell type or via paracrine signaling. Accumulating evidence has shown that ERα and PR are expressed in cell populations that do not totally overlap. *GJA1* is usually associated with basal cells while PR is thought to be expressed mainly in hormone-responsive luminal cell [1]. However, PR has been detected in some human breast basal cells, especially within immature lobules [1], suggesting an expression in primitive basal progenitor cells. PR has been suggested to coordinate basal cell proliferation, either via paracrine or autocrine stimulation [1]. It was also reported that the unliganded progesterone receptor isoform A (PRA) could activate Cx43 transcription by interacting with AP-1 heterodimers composed of FRA2 and JUND [42]. More studies are needed to better understand Cx43 localization and regulation, as well as its potential link with hormones. This knowledge is essential to further understand mammary gland morphogenesis and how Cx43 and hormones are involved in breast cancer.

Several other questions remain unanswered regarding the link between *GJA1* and ERα, PR and HER2. While the receptor’s protein and mRNA levels were well correlated in our study, their functional status in the samples is unknown. Protein expression data for some phosphorylated forms of ER (ERα_pS118) and HER2 (HER2_pY1248) receptor were available. Beyond single phosphorylation, the activation of these receptors is mostly dependent on complex post-transcriptional processing which affects receptors’ specific functions and gene transcription. As a result, prognostic significance of ERα has been shown to be phosphorylation site-specific [46]. Therefore, it cannot be excluded that *GJA1* mRNA expression can be regulated by ERα or HER2 and that these links could not be captured by expression profiles from breast cancer samples. Regardless of the precise nature of the link between *GJA1* and hormone receptors, our results suggest that *GJA1* level is dependent on the overall molecular context provided by each breast cancer subtype and that this might relate to *PGR* level, at least in some subtypes.

### 3.3. Upregulation of Cx43 mRNA Is Not Driven by DNA Amplification in Breast Cancer

Somatic DNA-level chromosomal aberrations are a defining characteristic of cancer and are common in breast carcinoma. Genomic loss and amplification cause decreases and increases in the transcription of genes in the region and often with concomitant effects of protein expression. We found that *GJA1* is rarely the target of such somatic events, and when it occurred it was often in Her2e and basal subtypes, consistent with the observation that these two subtypes generally have an increased amount of genomic instability in comparison to the luminal subtypes. Our results are also in accordance with previous studies that have shown that the region of human chromosome 6 where *GJA1* is located (6q22.31) has a relatively low level of amplification and deletions [47]. Cx43 was rarely mutated in breast cancer samples. Together, these results suggest that *GJA1* dysregulation at the mRNA and protein levels involves a dysregulation of other factors impacting the transcription (epigenetics, transcription factors) or mRNA stability.

### 3.4. Cx43 mRNA Level Is Dysregulated at the Early Stages of Breast Cancer

It was previously reported that, in primary tumors, Cx43 protein expression correlated with clinical stages [45]. Our analysis of microarray data from large cohorts of primary tumors suggested that Cx43 is decreased in a subset of tumors during early carcinogenesis (stage 0) and is re-expressed at higher levels in stage I tumors. While stage 0 of the luminal subtypes showed an increased variance of expression, those of basal-like and Her2e tumors had a significantly reduced expression compared to normal tissues. However, we could not observe a robust mRNA reduction, or increase, in later stages compared either to early tumor stages or to normal tissues.

A previous study investigated immunohistochemistry for Cx43 protein expression in ductal carcinoma in situ (DCIS), DCIS with microinvasion, DCIS with invasive ductal carcinoma (IDC) and IDC alone. In pooled tumors as well as in most subtypes, the lowest expression of Cx43 protein occurred neither in DCIS nor IDC alone but precisely in DCIS with microinvasion where only three out of thirty-seven cases (8%) were positive [36]. On the other side, out of 193 invasive lesions, sixty-three (33%) expressed Cx43. When looking specifically at the Her2e subtype, Cx43 was not expressed in a lower number of DCIS with microinvasion as in other subtypes since Cx43 was rarely expressed. Cx43 was present in only one of twenty IDC samples while the remaining twenty-six samples of other groups (DCIS, DCIS with microinvasion and DCIS with IDC) were all negatives. Whether or not the stromal compartment was included in the analysis was not specified. These results are consistent with our observation that, in all subtypes, DCIS (typically stage 0) had a lower expression than invasive stage I tumors. Together, these result point to Cx43 dysregulation as an early event in tumorigenesis, similar to what has been observed in the early stages of cervix, endometrial and thyroid cancers [48].

Moreover, it should be noted that while breast cancer stages are based on the size and the spreading of the disease in the tissue or to distant sites, the mRNA expression profiles we used only account for gene expression in whole primary tumors. Important morphologic information is therefore lost. During cancer progression, localized neoplastic cells acquire the capacity to invade surrounding tissues, and eventually reach the blood or lymphatic vasculature, allowing them to spread to other organs [49]. Depending on the stage and their location within the tumor or the tissue, these tumor cell populations face different challenges depending on the processes accomplished and on the microenvironment surrounding them [49]. Microarray data do not make it possible to either finely assess the expression of specific cells according to their specific localization in the tumor or to distinguish tumor gene expression from the stroma. A comprehensive study of events occurring early in carcinogenesis and accounting for the geographical localization within the tumors at primary or distant sites, and for the different cell populations in a subtype-dependent manner, is therefore the next logical step in further understanding Cx43’s role in tumor progression.

### 3.5. The Apparent Grade-Dependent Decrease in Cx43 Is Linked to Its Low Expression in More Aggressive Subtypes

Tumor grade is a measure of the degree of abnormality of tumor cells and of dedifferentiation of cancer tissues compared to normal breast tissue. Our results showed that, when all tumors are pooled, the *GJA1* mRNA level seems to increase in grade 1 tumors compared to normal tissues and gradually decrease with increasing grade. However, stratifying the tumors by subtype showed that within an intrinsic subtype, the distribution of the tumors within each grade varies considerably. Indeed, luminal A and B tumors are more frequently of grade 1 or 2 and some of them over-express *GJA1* (Figure 2), while most basal and Her2e tumors, that express a low level of Cx43, are mostly of grade 3. As a result, *GJA1* mRNA is not lost with grade in individual subtypes. These results suggest that the observed correlation in pooled tumors is, in fact, a bias attributable to the pooling of the tumors, and reflects the high grade of basal and Her2e tumors. These results also highlight how pooling the different intrinsic subtypes, expressing varying degrees of *GJA1*, can introduce important biases in cohort analysis and will likely yield different results depending on the composition, in terms of the subtypes, of the cohorts studied.

### 3.6. High Expression of Cx43 Is Associated with a Good Prognostic in Luminal Subtypes, but with a Worse Prognostic in Her2e Tumors

Our results showed that, consistent with its ascribed role as a tumor suppressor in breast cancer, Cx43 was expressed at lower levels in more aggressive basal and Her2e subtypes than in luminal subtypes. Survival analysis of pooled tumors therefore showed a better survival of tumors highly expressing Cx43. However, as with grade, pooling breast cancer subtypes to analyze the effect of *GJA1* on the outcome introduces biases. Tumors expressing low levels of *GJA1* are overrepresented in aggressive basal and Her2e tumors, likely dragging down the survival of the group expressing a low level of *GJA1*. Therefore, performing survival analysis on pooled tumors, a good prognosis patient is automatically segregated into the curve of tumors highly expressing *GJA1*, and vice versa.

Paradoxically, the prognostic associated with Cx43 expression diverged depending on the intrinsic subtype, with a good prognosis in luminal tumors and an opposite trend in Her2e tumors. A previous study using immunohistochemistry found no correlation between Cx43 protein level and patient outcome [44]. However, similar to our results, more recent studies using expression array-based survival curves found that a high *GJA1* expression was associated with a better prognosis in ERα-positive breast cancer tumors, while an opposite trend was observed in ERα-negative tumors [33] and Her2e tumors [34]. The worse prognosis associated with *GJA1* in Her2e tumors suggests that *GJA1* function in breast cancer might not just be tissue- and stage-dependent, as suggested by others [11,17], but might also be subtype-dependent.

Cx43 has been reported to be expressed both in epithelial and stromal cells types. The molecular landscape provided by different cell types and/or by different breast cancer subtypes might provide different context, possibly allowing Cx43 to assume different functions and leading to different outcomes. It could be hypothesized that such context may provide different sets of interacting partners for *GJA1*, and its expression might even be driven by a different set of transcriptional or epigenetic regulators. In addition, an important determinant of the capacity of Cx43 to assume its channel function is unarguably its proper membrane localization. From array based mRNA expression data, it is until now impossible to assess neither the cellular localization nor the functional status of Cx43. It is very likely that these important and relevant information would contribute to a more complete understanding of the functions of Cx43 in breast cancer. For instance, a recent study demonstrated that over-expressing Cx43 in two different HER2-positive breast cancer cell lines lead to a diverging ability to proliferate, migrate, form mammospheres and form tumors in mice. Tumorigenic characteristics of the cancer cells were enhanced when functional gap junction channels could not be formed upon Cx43 over-expression, but were reduced when membrane gap junctions plaques allowed cells to communicate [34]. These aspects of Cx43 biology might explain its different roles according to subtypes but also possibly within subtypes and should therefore be addressed. Additional researches are required to better understand the context that allows Cx43 to suppress or promote carcinogenesis in different intrinsic subtypes.

## 4. Materials and Methods

### 4.1. Gene Expression

We used 4K samples over different expression platforms. Vanvliet used Affymetrix Human Genome U133A (data processed with Robust Multi-Array Average (RMA)) [50]. Curtis discovery and Curtis validation used the Illumina HT-12 v3 platform (expression given as a Log2 intensity level) [47]. The Cancer Genome Atlas (TCGA) used a custom Agilent G4502A 244K array (expression given as Log2 Lowess normalized ratio) [51]. NKI used a Hu25K Agilent platform (samples were hybridized against a pool of equal amount of RNA from each patient and gene expression is given as a log10 of intensity ratio) [52]. Normalized signal per probe or probe set mRNA expression was downloaded for tumor samples for all five datasets. Breast cancer intrinsic subtype was assigned to each sample with the Pam50 molecular subtyping algorithm using the R genefu package [29]. Survival for each case was determined as in [27].

ERα, PR and HER2 status provided in the original publication of the dataset was used. For TCGA, ER, PR and HER2 status was obtained by immunohistochemistry (IHC). For NKI, ER and PR status was determined by IHC and the sample was considered positive if at least 10% of the cells were positive. For Vanvliet, ER and PR status was determined with the Bioconductor package ROCR based on the expression of the probe 205225_at and validated with IHC when available. NKI and Vanvliet HER2 status was determined using the probes of the HER2 amplicon genes. For Curtis datasets, ER, PR and HER2 status was based on mRNA expression. In the TCGA dataset, the level of some proteins has been investigated with reverse phase protein assay (RPPA). Data were available for total ERα, PR and HER2 as well as for the phosphorylated forms of ERα (pS118) and HER2 (pY1248) that are at least partially indicative of the activation status [46,53]. *GJA1* protein level obtained by mass spectrometry for 105 TCGA samples was retrieved from the protein report found at the Clinical Proteomic Tumor Analysis Consortium (CPTAC) data portal [54]. Levels are given as the log2 of the ratio of each sample with respect to a pooled reporter sample.

### 4.2. DNA Alteration

Copy number alterations (CNAs) were measured in the TCGA dataset with Affymetrix 6.0 single nucleotide polymorphism (SNP) arrays and segmented using Circular Binary Segmentation (labeled here as Relative linear copy number values) [51]. Data were further processed by TCGA using GISTIC 2.0 to assign the Putative copy number calls per gene (−2: Homozygous deletion, −1: Hemizygous deletion, 0: Neutral/no change, 1: Gain, 2: High level amplification) [51]. Mutations were detected using whole-exome sequencing after controlling for germline and normal adjacent tissue mutations [51]. Linear and called CNA data as well as mutation data for the TCGA dataset were retrieved using R via cBioportal [55,56]. A total of 977 patients had data for mutations [51].

### 4.3. Survival Analysis

Survival data was available for all five datasets. The log-rank test was used to estimate significance and hazard ratios (95% CI) were computed via Cox regression using survival package [57]. ROC curves were computed using the pROC package [58]. Kaplan–Meier plots were used to visualize the data. The best cutoff to determine tumors expressing high or low levels of Cx43 was selected using either the ROC curves or based on the smallest *p* value of the log rank test computed for each threshold between 10 and 90. For aggregated datasets analysis, each cohort was first split into groups based on the selected threshold and datasets were pooled only after splitting.

In addition, survival analyses were computed using the BreastMark and KMPlotter web platforms that use several well-known dataset [35,59]. BreastMark allows thresholds of 25, 50 and 75 percentile to be selected to split the different cohorts used before aggregating them. For each analysis, we selected the threshold giving the best results. Since less samples were available in KMPlotter for statistical computation, we only included analyses for which there were at least 100 samples to draw both high and low expression curves for each subtype.

### 4.4. Statistical Analysis

All statistical analyses were carried out with R version 3.4.3 [60]. For two class comparisons, (cancer vs. normal tissues; positive vs. negative hormonal status) the Wilcoxon-Mann-Witney test was used. When more than two classes were compared, the Kruskall-Wallis test was used followed by the Dunn post-hoc test to assess the statistical significance for each pair of samples (to compare subtypes). For stages and grades, differential gene expression was assessed using Limma package [61]. A Benjamini-Hochberg correction was applied to adjust the *p* values for multiple testing. Because each subtype had a different number of patients, when correlation tests were performed between the expression of *GJA1* and the expression of other genes, a non-parametric bootstrap procedure was used for each subtype to derive the mean correlation coefficient and a percent confidence interval.

## 5. Conclusions

Our study has clarified the expression pattern of *GJA1* mRNA in breast cancer and showed that *GJA1* expression, as well as its prognostic significance, is dependent on breast cancer subtype. We also highlighted important biases that are introduced in analyzing pooled tumors. These biases need to be taken into consideration when studying *GJA1*, but also numerous other genes that are known to be linked, for instance, to ERα expression. Breast cancers are heterogeneous and genetically diverse and the lack of recognition of this molecular heterogeneity might explain the conflicting results from the literature, not only for *GJA1*, but potentially for other tumor suppressors or oncogenes. Overall, these results clearly showed that the molecular context where Cx43 is expressed in general, and the tumor subtypes of breast cancer in particular, should be taken into account when investigating Cx43’s role in carcinogenesis.

## Abbreviations

CNA	Copy number alterations
Cx43	Connexin 43
DCIS	Ductal carcinoma in situ
ERα	Estrogen receptor alpha
*ERBB2*	erb-b2 receptor tyrosine kinase 2
GJIC	Gap Junction Intercellular Communication
*GJA1*	Gap Junction protein alpha 1, the gene encoding Connexin 43
Her2e	HER2-enriched
IDC	Invasive ductal carcinoma
LumA	Luminal A
LumB	Luminal B
PR	Progesterone Receptor
RPPA	Reverse phase protein assay
TCGA	The Cancer Genome Atlas

## References

[1] Arendt L.M., Kuperwasser C. (2015). Form and function: how estrogen and progesterone regulate the mammary epithelial hierarchy. J. Mammary Gland Biol. Neoplasia.

[2] Su V., Lau A.F. (2014). Connexins: Mechanisms regulating protein levels and intercellular communication. FEBS Lett..

[3] Plante I., Stewart M.K., Barr K., Allan A.L., Laird D.W. (2011). Cx43 suppresses mammary tumor metastasis to the lung in a Cx43 mutant mouse model of human disease. Oncogene.

[4] Dianati E., Poiraud J., Weber-Ouellette A., Plante I. (2016). Connexins, E-cadherin, Claudin-7 and beta-catenin transiently form junctional nexuses during the post-natal mammary gland development. Dev. Biol..

[5] Monaghan P., Clarke C., Perusinghe N.P., Moss D.W., Chen X.Y., Evans W.H. (1996). Gap junction distribution and connexin expression in human breast. Exp. Cell Res..

[6] Talhouk R.S., Elble R.C., Bassam R., Daher M., Sfeir A., Mosleh L.A., El-Khoury H., Hamoui S., Pauli B.U., El-Sabban M.E. (2005). Developmental expression patterns and regulation of connexins in the mouse mammary gland: Expression of connexin30 in lactogenesis. Cell Tissue Res..

[7] Pitelka D.R., Hamamoto S.T., Duafala J.G., Nemanic M.K. (1973). Cell contacts in the mouse mammary gland. I. Normal gland in postnatal development and the secretory cycle. J. Cell Biol..

[8] Pollmann M.A., Shao Q., Laird D.W., Sandig M. (2005). Connexin 43 mediated gap junctional communication enhances breast tumor cell diapedesis in culture. Breast Cancer Res..

[9] Tomasetto C., Neveu M.J., Daley J., Horan P.K., Sager R. (1993). Specificity of gap junction communication among human mammary cells and connexin transfectants in culture. J. Cell Biol..

[10] Woodward T.L., Sia M.A., Blaschuk O.W., Turner J.D., Laird D.W. (1998). Deficient epithelial-fibroblast heterocellular gap junction communication can be overcome by co-culture with an intermediate cell type but not by E-cadherin transgene expression. J. Cell Sci..

[11] Naus C.C., Laird D.W. (2010). Implications and challenges of connexin connections to cancer. Nat. Rev. Cancer.

[12] Laird D.W., Fistouris P., Batist G., Alpert L., Huynh H.T., Carystinos G.D., Alaoui-Jamali M.A. (1999). Deficiency of connexin43 gap junctions is an independent marker for breast tumors. Cancer Res..

[13] Lee S.W., Tomasetto C., Paul D., Keyomarsi K., Sager R. (1992). Transcriptional downregulation of gap-junction proteins blocks junctional communication in human mammary tumor cell lines. J. Cell Biol..

[14] Gould V.E., Mosquera J.M., Leykauf K., Gattuso P., Durst M., Alonso A. (2005). The phosphorylated form of connexin43 is up-regulated in breast hyperplasias and carcinomas and in their neoformed capillaries. Hum. Pathol..

[15] Jamieson S., Going J.J., D’Arcy R., George W.D. (1998). Expression of gap junction proteins connexin 26 and connexin 43 in normal human breast and in breast tumours. J. Pathol..

[16] Kanczuga-Koda L., Sulkowska M., Koda M., Reszec J., Famulski W., Baltaziak M., Sulkowski S. (2003). Expression of connexin 43 in breast cancer in comparison with mammary dysplasia and the normal mammary gland. Folia Morphol. (Warsz).

[17] El-Saghir J.A., El-Habre E.T., El-Sabban M.E., Talhouk R.S. (2011). Connexins: A junctional crossroad to breast cancer. Int. J. Dev. Biol..

[18] Nicolson G.L., Dulski K.M., Trosko J.E. (1988). Loss of intercellular junctional communication correlates with metastatic potential in mammary adenocarcinoma cells. Proc. Natl. Acad. Sci. USA.

[19] Ren J., Hamada J., Takeichi N., Fujikawa S., Kobayashi H. (1990). Ultrastructural differences in junctional intercellular communication between highly and weakly metastatic clones derived from rat mammary carcinoma. Cancer Res..

[20] Li Z., Zhou Z., Welch D.R., Donahue H.J. (2008). Expressing connexin 43 in breast cancer cells reduces their metastasis to lungs. Clin. Exp. Metastasis.

[21] Qin H., Shao Q., Curtis H., Galipeau J., Belliveau D.J., Wang T., Alaoui-Jamali M.A., Laird D.W. (2002). Retroviral delivery of connexin genes to human breast tumor cells inhibits in vivo tumor growth by a mechanism that is independent of significant gap junctional intercellular communication. J. Biol. Chem..

[22] El Sabban M.E., Pauli B.U. (1994). Adhesion-mediated gap junctional communication between lung-metastatatic cancer cells and endothelium. Invasion Metastasis.

[23] Elzarrad M.K., Haroon A., Willecke K., Dobrowolski R., Gillespie M.N., Al-Mehdi A.B. (2008). Connexin-43 upregulation in micrometastases and tumor vasculature and its role in tumor cell attachment to pulmonary endothelium. BMC Med..

[24] Stoletov K., Strnadel J., Zardouzian E., Momiyama M., Park F.D., Kelber J.A., Pizzo D.P., Hoffman R., Vandenberg S.R., Klemke R.L. (2013). Role of connexins in metastatic breast cancer and melanoma brain colonization. J. Cell Sci..

[25] Kanczuga-Koda L., Sulkowski S., Lenczewski A., Koda M., Wincewicz A., Baltaziak M., Sulkowska M. (2006). Increased expression of connexins 26 and 43 in lymph node metastases of breast cancer. J. Clin. Pathol..

[26] Sorlie T., Perou C.M., Tibshirani R., Aas T., Geisler S., Johnsen H., Hastie T., Eisen M.B., van de Rijn M., Jeffrey S.S. (2001). Gene expression patterns of breast carcinomas distinguish tumor subclasses with clinical implications. Proc. Natl. Acad. Sci. USA.

[27] Tofigh A., Suderman M., Paquet E.R., Livingstone J., Bertos N., Saleh S.M., Zhao H., Souleimanova M., Cory S., Lesurf R. (2014). The prognostic ease and difficulty of invasive breast carcinoma. Cell Rep..

[28] Uhlen M., Bjorling E., Agaton C., Szigyarto C.A., Amini B., Andersen E., Andersson A.C., Angelidou P., Asplund A., Asplund C. (2005). A human protein atlas for normal and cancer tissues based on antibody proteomics. Mol. Cell Proteom..

[29] Gendoo D.M., Ratanasirigulchai N., Schroder M.S., Pare L., Parker J.S., Prat A., Haibe-Kains B. (2016). Genefu: An R/Bioconductor package for computation of gene expression-based signatures in breast cancer. Bioinformatics.

[30] Mitra S., Annamalai L., Chakraborty S., Johnson K., Song X.H., Batra S.K., Mehta P.P. (2006). Androgen-regulated formation and degradation of gap junctions in androgen-responsive human prostate cancer cells. Mol. Biol. Cell.

[31] Ren J., Wang X.H., Wang G.C., Wu J.H. (2013). 17beta estradiol regulation of connexin 43-based gap junction and mechanosensitivity through classical estrogen receptor pathway in osteocyte-like MLO-Y4 cells. Bone.

[32] Stewart M., Simek J., Laird D. (2015). Insights into the role of Connexins in Mammary Gland Morphogenesis and Function. Reproduction.

[33] Teleki I., Szasz A.M., Maros M.E., Gyorffy B., Kulka J., Meggyeshazi N., Kiszner G., Balla P., Samu A., Krenacs T. (2014). Correlations of differentially expressed gap junction connexins cx26, cx30, cx32, cx43 and cx46 with breast cancer progression and prognosis. PLoS ONE.

[34] Yeh E.S., Williams C.J., Williams C.B., Bonilla I.V., Klauber-DeMore N., Phillips S.L. (2017). Dysregulated connexin 43 in HER2-positive drug resistant breast cancer cells enhances proliferation and migration. Oncotarget.

[35] Gyorffy B., Lanczky A., Eklund A.C., Denkert C., Budczies J., Li Q., Szallasi Z. (2010). An online survival analysis tool to rapidly assess the effect of 22,277 genes on breast cancer prognosis using microarray data of 1809 patients. Breast Cancer Res. Treat..

[36] Park S.Y., Lee H.E., Li H., Shipitsin M., Gelman R., Polyak K. (2010). Heterogeneity for Stem Cell-Related Markers According to Tumor Subtype and Histologic Stage in Breast Cancer. Clin. Cancer Res..

[37] Fu Y., Shao Z.M., He Q.Z., Jiang B.Q., Wu Y., Zhuang Z.G. (2015). Hsa-miR-206 represses the proliferation and invasion of breast cancer cells by targeting Cx43. Eur. Rev. Med. Pharmacol. Sci..

[38] Clarke C., Madden S.F., Doolan P., Aherne S.T., Joyce H., O’Driscoll L., Gallagher W.M., Hennessy B.T., Moriarty M., Crown J. (2013). Correlating transcriptional networks to breast cancer survival: A large-scale coexpression analysis. Carcinogenesis.

[39] Yoshihara K., Shahmoradgoli M., Martinez E., Vegesna R., Kim H., Torres-Garcia W., Trevino V., Shen H., Laird P.W., Levine D.A. (2013). Inferring tumour purity and stromal and immune cell admixture from expression data. Nat. Commun..

[40] Grummer R., Traub O., Winterhager E. (1999). Gap junction connexin genes cx26 and cx43 are differentially regulated by ovarian steroid hormones in rat endometrium. Endocrinology.

[41] Gulinello M., Etgen A.M. (2005). Sexually dimorphic hormonal regulation of the gap junction protein, CX43, in rats and altered female reproductive function in CX43+/− mice. Brain Res..

[42] Nadeem L., Shynlova O., Matysiak-Zablocki E., Mesiano S., Dong X., Lye S. (2016). Molecular evidence of functional progesterone withdrawal in human myometrium. Nat. Commun..

[43] Yu J., Berga S.L., Johnston-MacAnanny E.B., Sidell N., Bagchi I.C., Bagchi M.K., Taylor R.N. (2016). Endometrial Stromal Decidualization Responds Reversibly to Hormone Stimulation and Withdrawal. Endocrinology.

[44] Conklin C., Huntsman D., Yorida E., Makretsov N., Turbin D., Bechberger J.F., Sin W.C., Naus C.C. (2007). Tissue microarray analysis of connexin expression and its prognostic significance in human breast cancer. Cancer Lett..

[45] Teleki I., Krenacs T., Szasz M.A., Kulka J., Wichmann B., Leo C., Papassotiropoulos B., Riemenschnitter C., Moch H., Varga Z. (2013). The potential prognostic value of connexin 26 and 46 expression in neoadjuvant-treated breast cancer. BMC Cancer.

[46] Murphy L.C., Seekallu S.V., Watson P.H. (2011). Clinical significance of estrogen receptor phosphorylation. Endocr. Relat. Cancer.

[47] Curtis C., Shah S.P., Chin S.F., Turashvili G., Rueda O.M., Dunning M.J., Speed D., Lynch A.G., Samarajiwa S., Yuan Y. (2012). The genomic and transcriptomic architecture of 2000 breast tumours reveals novel subgroups. Nature.

[48] Mesnil M., Crespin S., Avanzo J.L., Zaidan-Dagli M.L. (2005). Defective gap junctional intercellular communication in the carcinogenic process. Biochim. Biophys. Acta.

[49] Valastyan S., Weinberg R.A. (2011). Tumor metastasis: Molecular insights and evolving paradigms. Cell.

[50] Van Vliet M.H., Reyal F., Horlings H.M., van de Vijver M.J., Reinders M.J., Wessels L.F. (2008). Pooling breast cancer datasets has a synergetic effect on classification performance and improves signature stability. BMC Genom..

[51] TCGA (2012). Comprehensive molecular portraits of human breast tumours. Nature.

[52] Van de Vijver M.J., He Y.D., van’t Veer L.J., Dai H., Hart A.A., Voskuil D.W., Schreiber G.J., Peterse J.L., Roberts C., Marton M.J. (2002). A gene-expression signature as a predictor of survival in breast cancer. N. Engl. J. Med..

[53] Hazan R., Margolis B., Dombalagian M., Ullrich A., Zilberstein A., Schlessinger J. (1990). Identification of autophosphorylation sites of HER2/neu. Cell Growth Differ..

[54] Mertins P., Mani D.R., Ruggles K.V., Gillette M.A., Clauser K.R., Wang P., Wang X., Qiao J.W., Cao S., Petralia F. (2016). Proteogenomics connects somatic mutations to signalling in breast cancer. Nature.

[55] Ciriello G., Gatza M.L., Beck A.H., Wilkerson M.D., Rhie S.K., Pastore A., Zhang H., McLellan M., Yau C., Kandoth C. (2015). Comprehensive Molecular Portraits of Invasive Lobular Breast Cancer. Cell.

[56] Gao J., Aksoy B.A., Dogrusoz U., Dresdner G., Gross B., Sumer S.O., Sun Y., Jacobsen A., Sinha R., Larsson E. (2013). Integrative analysis of complex cancer genomics and clinical profiles using the cBioPortal. Sci. Signal..

[57] Therneau T.M., Grambsch P.M. (2000). Modeling Survival Data: Extending the Cox Model.

[58] Robin X., Turck N., Hainard A., Tiberti N., Lisacek F., Sanchez J.C., Muller M. (2011). pROC: An open-source package for R and S+ to analyze and compare ROC curves. BMC Bioinform..

[59] Madden S.F., Clarke C., Gaule P., Aherne S.T., O’Donovan N., Clynes M., Crown J., Gallagher W.M. (2013). BreastMark: An integrated approach to mining publicly available transcriptomic datasets relating to breast cancer outcome. Breast Cancer Res..

[60] R Core Team (2016). R: A Language and Environment for Statistical Computing.

[61] Ritchie M.E., Phipson B., Wu D., Hu Y., Law C.W., Shi W., Smyth G.K. (2015). limma powers differential expression analyses for RNA-sequencing and microarray studies. Nucl. Acids Res..

